# Effects of Vegetation Restoration on Soil Erosion on the Loess Plateau: A Case Study in the Ansai Watershed

**DOI:** 10.3390/ijerph18126266

**Published:** 2021-06-10

**Authors:** Hui Wei, Wenwu Zhao, Han Wang

**Affiliations:** 1 State Key Laboratory of Earth Surface Processes and Resource Ecology, Faculty of Geographical Science, Beijing Normal University, Beijing 100875, China; irene1993weihui@163.com (H.W.); hanwang_226@163.com (H.W.); 2 Institute of Land Surface System and Sustainable Development, Faculty of Geographical Science, Beijing Normal University, Beijing 100875, China

**Keywords:** vegetation restoration, soil erosion, land use, Loess Plateau, Ansai Watershed

## Abstract

Large-scale vegetation restoration greatly changed the soil erosion environment in the Loess Plateau since the implementation of the “Grain for Green Project” (GGP) in 1999. Evaluating the effects of vegetation restoration on soil erosion is significant to local soil and water conservation and vegetation construction. Taking the Ansai Watershed as the case area, this study calculated the soil erosion modulus from 2000 to 2015 under the initial and current scenarios of vegetation restoration, using the Chinese Soil Loess Equation (CSLE), based on rainfall and soil data, remote sensing images and socio-economic data. The effect of vegetation restoration on soil erosion was evaluated by comparing the average annual soil erosion modulus under two scenarios among 16 years. The results showed: (1) vegetation restoration significantly changed the local land use, characterized by the conversion of farmland to grassland, arboreal land, and shrub land. From 2000 to 2015, the area of arboreal land, shrub land, and grassland increased from 19.46 km^2^, 19.43 km^2^, and 719.49 km^2^ to 99.26 km^2^, 75.97 km^2^, and 1084.24 km^2^; while the farmland area decreased from 547.90 km^2^ to 34.35 km^2^; (2) the average annual soil erosion modulus from 2000 to 2015 under the initial and current scenarios of vegetation restoration was 114.44 t/(hm²·a) and 78.42 t/(hm²·a), respectively, with an average annual reduction of 4.81 × 10^6^ t of soil erosion amount thanks to the vegetation restoration; (3) the dominant soil erosion intensity changed from “severe and light erosion” to “moderate and light erosion”, vegetation restoration greatly improved the soil erosion environment in the study area; (4) areas with increased erosion and decreased erosion were alternately distributed, accounting for 48% and 52% of the total land area, and mainly distributed in the northwest and southeast of the watershed, respectively. Irrational land use changes in local areas (such as the conversion of farmland and grassland into construction land, etc.) and the ineffective implementation of vegetation restoration are the main reasons leading to the existence of areas with increased erosion.

## 1. Introduction

Approximately 20% of the land area is currently experiencing a decline in productivity linked to erosion, wastage, and pollution in the world [1]. Among these factors, soil erosion not only causes problems such as soil quality decline, land degradation, and loss of farmland resources, but also leads to a series of ecological and environmental problems such as water environment deterioration, river siltation, debris flows, and even flood disasters [2,3,4,5,6]. The global soil erosion area has reached 25 million km^2^, accounting for 16.8% of the total land area and threatening the security of 27% of the total farmland area [7]. To this end, soil erosion has become a global ecological and environmental problem [8,9,10,11,12]. The land that has undergone water erosion or wind erosion is up to 3 million km^2^ in China, accounting for approximately 32% of the total land area [13]. The Loess Plateau is the region with the most severe soil erosion in China, where the area of soil and water loss is as high as 4.5 × 10^5^ km^2^, mainly dominated by intensive erosion (>5000 t/(hm^2^·a)), and the average sediment transport over years is 1.6 × 10^9^ t [14]. The area, intensity, and amounts of the soil erosion in the Loess Plateau are the largest in the world [14,15,16].

To effectively control soil erosion and ecological degradation, the Chinese government implemented the “Grain for Green Project” (GGP) since 1999 to return farmland with slopes of 25° or more to perennial vegetation [2,17,18]. Vegetation restoration triggered by the “GGP” is an effective approach to ecological construction and soil erosion control in the western region of China [19,20,21]. Since the implementation of the “GGP”, the soil erosion environment in the Loess Plateau has been greatly changed by large-scale vegetation restoration [2,9,17,18,19]. The United Nations General Assembly announced the “United Nations Decade on Ecosystem Restoration 2021–2030 (UNDER)” on 1 March 2019, a movement aimed to expand the restoration of degraded and damaged ecosystems as an effective measure to address the climate crisis and enhance food security, water resources, and biodiversity [1,22]. Under the global background of the UNDER, assessing the effects of vegetation restoration on soil erosion over the past 20 years is significant to sustaining the water and soil conservation benefits of vegetation restoration in the Loess Plateau.

Selecting the Ansai Watershed as the case study area of the Loess Plateau, this study identified the effects of vegetation restoration on soil erosion by comparing the differences between the soil erosion modulus from 2000 to 2015 under two land use scenarios (the initial and current scenarios of vegetation restoration). The research results have important theoretical and practical significance for regional soil and water conservation and vegetation construction.

## 2. Materials and Methods

### 2.1. Study Area

**Figure 1:**
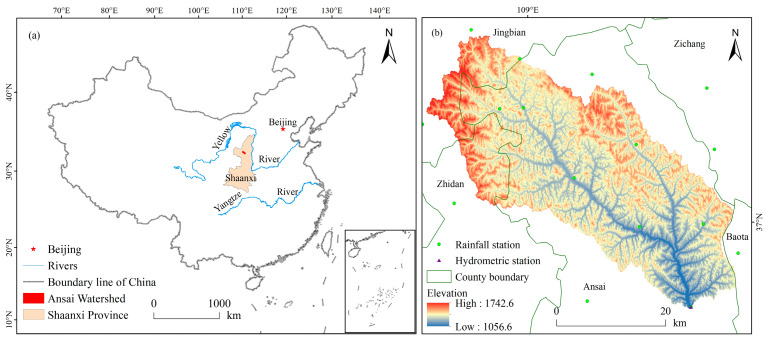
The study area: (**a**) The geographical location of the Ansai Watershed; (**b**) The elevation, rainfall and hydrometric stations distribution in the Ansai Watershed.

The Ansai watershed (108°5′44″–109°26′18″ E, 36°30′45″–37°19′3″ N) is located in the upper reaches of the Yanhe River basin, in the inland hinterland of the northwestern Loess Plateau. This watershed lies in the northern part of Shaanxi Province and borders the Ordos basin (Figure 1). It belongs to the typical loess hilly and gully region, and covers a total area of 1334.00 km^2^ [23]. The soil type in the study area is loess soil, with low fertility and high vulnerability to erosion [24,25]. The topography is complex and varied, and the land surface is fragmented into different land uses, dominated by rain-fed farmland, grassland, shrubland, and forest land [26]. The elevations within the watershed are low in the southeast and high in the northwest, ranging between 997 m and 1731 m above sea level [23]. The climate is a continental semi-arid monsoon climate in the middle temperate zone, and the average annual precipitation is 505.3 mm, and 74% of the rainfall occurs from June to September [26].

### 2.2. Data Sources

**Figure 2:**
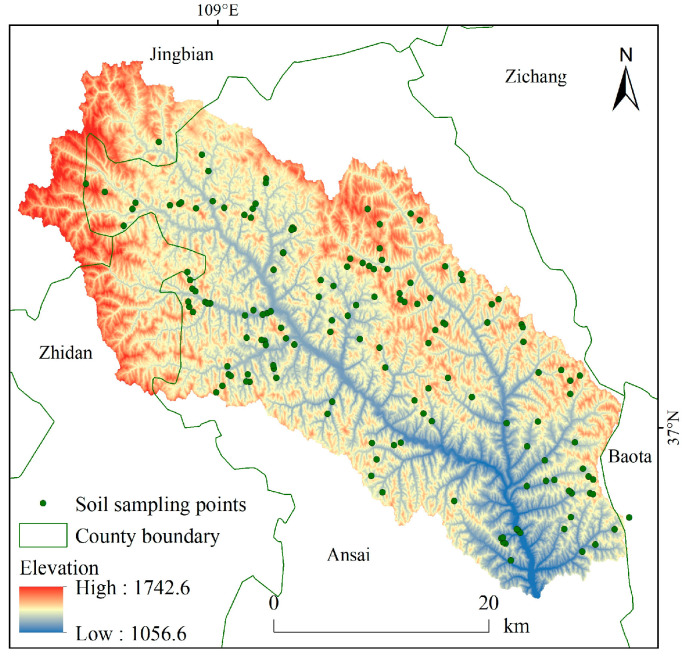
Soil sampling points in the Ansai Watershed.

We used 25 m resolution DEM data, obtained from the 1:50,000 database of the National Center for Basic Geographic Information of China [27]. Vector land cover data in 2000 and 2015 was obtained from the Data Center for Resources and Environmental Sciences at the Chinese Academy of Sciences [28]. Daily rainfall data at 20 rainfall stations in and around the Ansai Watershed from 2000 to 2015 was collected from the *Hydrological Yearbook of the People’s Republic of China* [29]. Remote sensing images from 2000 to 2015 were obtained from the Geospatial Data Cloud [30]. Terrace and silting dam data from 2000 to 2015 were collected from the *Statistical Yearbook of Ansai County*. Soil data, derived from a dataset of 151 sample points was obtained from a soil survey in the Ansai Watershed conducted in July to August of 2014. In Figure 2, 151 soil sample points are evenly distributed in the Ansai watershed, which can well represent the soil attribute conditions in the study area; the location of sample points is accurately located by handheld GPS.

### 2.3. Research Methods

Since the 1980s, Chinese scholars proposed some regional models for soil erosion estimation based on the Universal Soil Loss Equation (USLE) and combined with local topographical features [31]. Among these models, the Chinese Soil Loss Equation (CSLE) fully considers the impact of biological, engineering, and tillage measures on the process and results of soil erosion, making it more suitable and widely used in the soil erosion estimation in China [32]. The CSLE model expression is as follows:

(1) A=R⋅K⋅L⋅S⋅B⋅E⋅T

where *A* is the average annual soil erosion modulus in t/(hm^2^·a); *R* is the rainfall erosivity factor in MJ·mm/(hm^2^·a); *K* is the soil erodibility factor in t·h/(MJ·mm); *L* and S are dimensionless factors of slope length and slope steepness, respectively; and *B*, *E*, *T* are dimensionless factors of biological-control, engineering-control, and tillage practices, respectively. The dimensionless factors of slope and soil conservation measures were defined as the ratio of soil erosion amounts from unit plot to actual plot with the aimed factor changed but the same sizes of other factors as the unit plot [32].

Based on the CSLE model and the control variable method, this study calculated the soil erosion modulus in the Ansai Watershed from 2000 to 2015 under two land use scenarios (the initial and current scenarios of vegetation restoration). The effect of vegetation restoration on soil erosion during the study period was identified by comparing the differences of average soil erosion modulus under two scenarios among 16 years. It should be noted that for the soil erosion modulus calculation under the two scenarios in the same year, the *R*, *K*, *L*, *S*, *E*, and *T* factors remained unchanged, while the *B* factor related to vegetation restoration was calculated based on the land use maps and remote sensing images in 2000 and 2015, respectively. Furthermore, the calculation method of each factor is as follows.

#### 2.3.1. Rainfall Erosivity (*R*) Factor

Rainfall erosivity (*R*) factor reflects the influence of rainfall on soil erosion [33]. In this study, we calculated the *R* factor according to the method proposed by Zhang et al. (2002) [34], a method that has been widely used in China [34,35]. The *R* factor, based on aggradations of half-month rainfall erosivity, was estimated using daily rainfall data obtained from the *Hydrological Yearbook of the People’s Republic of China* from 2000 to 2015. The calculation method is as follows:

(2) $$M_{i}=\alpha \overset{k}{\underset{j=1}{\sum }}{\left(D_{j}\right)}^{\beta }$$

where *M_i_* is the half-month rainfall erosivity in MJ·mm/(hm^2^·h·a), *k* refers to the number of days in a half-month, and *D_j_* represents the effective rainfall for day *j* in one half-month. *D_j_* is equal to the actual rainfall if the actual rainfall is greater than the threshold value of 12 mm, which is the standard for China’s erosive rainfall. Otherwise, *D_j_* is equal to zero [34]. The terms *α* and *β* are the undetermined parameters of the model and are calculated as follows:

(3) $$\beta =0.8363+\frac{18.177}{{\bar{P}}_{d12}}+\frac{24.455}{{\bar{P}}_{y12}}$$

(4) $$\alpha =21.586\beta^{-7.1891}$$

where ${\bar{P}}_{d12}$ is the daily average rainfall that is greater than 12 mm, and ${\bar{P}}_{y12}$ is the yearly average rainfall for days with rainfall more than 12 mm.

#### 2.3.2. Soil Erodibility (*K*) Factor

Soil erodibility (*K*) factor indicates both the susceptibility of soil to erosion and the amount and rate of runoff, as measured under standard plot conditions [36]. Previous studies found that the existing foreign *K* factor estimation models cannot be directly applied to the *K* factor calculation in China, and their estimated values are far greater than the actual measured values, while there is a certain linear relationship among them [37]. To this end, based on soil data obtained from the soil survey conducted in the Ansai Watershed, the *K* factor was calculated according to the Equations (5) and (6) [37,38].

(5) $$K_{shirazi}=7.594\left\{0.0017+0.0494e^{-\frac{1}{2}{\left[\frac{\log(D_{g})+1.675}{0.6986}\right]}^{2}}\right\}$$

(6) $$K=-0.00911+0.55066K_{shirazi}$$

where *D*_g_ is the geometric mean diameter of soil grains, and *K_shirazi_* is the *K* value estimated by the Equation (5) proposed by Shirazi et al. (1988) [38].

#### 2.3.3. Slope Length (*L*) and Steepness (*S*) Factor

Topography is an important factor that directly affects soil erosion. The slope length factor (*L*) and slope steepness factor (*S*) represent the effects of slope length and slope gradient on soil erosion, respectively [39]. The *L* factor and *S* factor can be calculated using the following equations:

(7) $$L={\left(\lambda /22.13\right)}^{m}$$

(8) m=β/(1+β)

(9) $$\beta =\left(\frac{\sin\theta }{0.0896}\right)/\left[3.0\times {\left(\sin\theta \right)}^{0.8}+0.56\right]$$

(10) $$S=\{\begin{array}{c} 10.8\times \sin\theta +0.03,\;\;\theta <5.14{}^{\circ}\\ 16.8\times \sin\theta -0.50,\;\;\theta \geq 5.14{}^{\circ} \end{array}$$

where *λ* is the length of the slope, *m* is the variable length-slope exponent, *β* is a factor that varies with slope gradient, and *θ* is slope gradient calculated based on DEM.

#### 2.3.4. Biological-Control (*B*) Factor

Biological-control (*B*) factor refers to the ratio of the soil erosion amounts of land with vegetation cover or field management, and that of continuously fallowed land under certain conditions [40,41]. In this study, we extracted NDVI values and calculated the vegetation coverage by using Equation (11) according to Li et al. (2020) [42] based on remote sensing images captured from June to September during 2000 to 2015; *B* factor was obtained according to the relationship between *B* factor and the land use types, and vegetation coverage (Table 1) [43]. The vegetation coverage was calculated as follows:

(11) $$f=\frac{NDVI-NDVI_{\min}}{NDVI_{\max}-NDVI_{\min}}$$

where *f* is the vegetation coverage, and *NDVI*_min_ and *NDVI*_max_ are the minimum and maximum *NDVI* values.

#### 2.3.5. Engineering-Control (*E*) Factor

Engineering-control (*E*) factor refers to the ratio of the soil erosion amounts occurring under certain engineering measures to that occurring without engineering measures under the same conditions [32]. The engineering-control practices in the Ansai Watershed mainly include silting dams and terraces. Considering the difficulty of collecting data on engineering measures, this study obtained terrace and silting dam data based on the *Statistical Yearbook of Ansai County* and calculated the *E* factor by referring to Equation (12) proposed by Xie et al. (2009) [44]:

(12) $$E=(1-\frac{S_{t}}{S}\times \alpha )(1-\frac{S_{d}}{S}\times \beta )$$

where *S_t_* is the terrace area, *S_d_* is the area controlled by silting dams, *S* is the total land area, and *α* and *β* refer to the sediment reduction coefficients of terrace and silting dam and are 0.836 and 1, respectively.

#### 2.3.6. Tillage (*T*) Factor

Tillage (*T*) factor refers to the ratio of the soil erosion amounts occurring under a specific tillage measure to that occurring under consistent flat cropping or slope tillage [45]. In this study, the slope gradient was extracted based on the DEM, and *T* factor was calculated according to the relationship between the slope gradient and the *T* factor (Table 2).

## 3. Results

### 3.1. Dynamic Land Use Changes Since Vegetation Restoration

**Figure 3:**
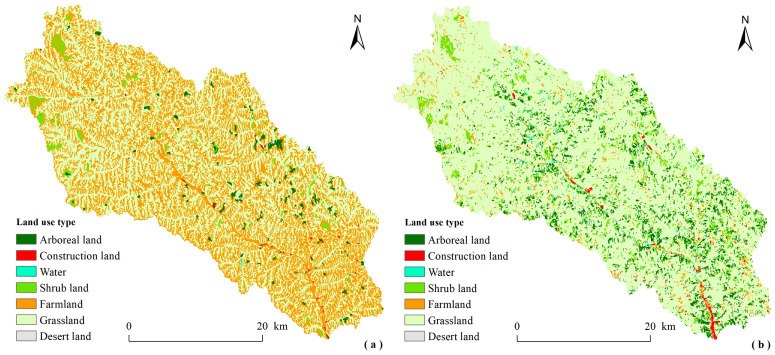
Land use map of the Ansai Watershed in 2000 (**a**) and in 2015 (**b**).

Large-scale vegetation restoration led to significant land use changes in the Ansai Watershed (Figure 3). Land use was dominated by grassland and farmland, while arboreal land and shrub land were scattered and did not form contiguous patterns in 2000. With the progress of vegetation restoration, grassland became the main land use type, and arboreal land and shrub land increased significantly and formed a distribution pattern which decreased gradually from southeast to northwest in 2015. Thanks to the relatively superior natural conditions and the location conditions closer to the urban area, compared with the upstream areas, the implementation of the “GGP” is more active and the benefits of vegetation restoration is more obvious in the downstream areas.

From 2000 to 2015, the area of arboreal land, shrub land, and grassland increased significantly in the Ansai Watershed, while the farmland decreased drastically, and the construction land, water land, and desert land increased slightly (Table 3). Furthermore, the farmland was mainly converted to grassland, followed by arboreal land, and shrub land; while a small part was converted to construction land, water land, and desert land. The primary driving factor of the changes was the implementation of “GGP” since 1999 [2,18,44].

### 3.2. Estimation of Soil Erosion under the Initial Scenario of Vegetation Restoration

The soil erosion modulus calculated based on the initial scenario of vegetation restoration was 31.18, 116.45, 170.88, 99.92, 147.21, 167.17, 88.56, 91.38, 55.03, 162.21, 80.11, 68.66, 115.97, 291.11, 115.96, and 31.19 t/(hm^2^·a) from 2000 to 2015, respectively, and the average soil erosion modulus among the 16 years was 114.56 t/(hm^2^·a) (Table 4). The light erosion accounted for the largest proportion, with 22.61%; the severe erosion followed, with an area of 300.67 km^2^; the areas of moderate erosion, extreme erosion, and serious erosion all exceeded 150 km^2^, accounting for 17.09%, 14.66%, and 12.29%, respectively, and the proportion of slight erosion was the smallest, with an area of 144.25 km^2^. It can be seen that the soil erosion under the initial scenario of vegetation restoration in the Ansai Watershed was dominated by severe erosion and light erosion, and the soil erosion situation was relatively severe.

### 3.3. Estimation of Soil Erosion under the Current Scenario of Vegetation Restoration

The soil erosion modulus calculated based on the current scenario of vegetation restoration was 20.88, 80.67, 119.51, 68.41, 101.07, 114.77, 60.17, 62.51, 36.16, 111.59, 54.80, 46.05, 80.01, 97.60, 79.13, and 21.44 t/(hm^2^·a) from 2000 to 2015, respectively, and the average soil erosion modulus among the 16 years was 78.42 t/(hm^2^·a) (Table 5). The light erosion accounted for 23.71% of the total area, covering the largest area of 316.30 km^2^; the moderate erosion and extreme erosion followed by 19.35% and 17.57%; the proportions of serious erosion and severe erosion all exceed 14%, with the area of 199.53 km^2^ and 188.68 km^2^, respectively, and the proportion of slight erosion was the smallest, with an area of 137.10 km^2^. Therefore, in contrast from the soil erosion dominated by severe and light erosion under the initial stage of vegetation restoration, soil erosion under the current scenario of vegetation restoration was dominated by light erosion and moderate erosion in the Ansai Watershed. Furthermore, the proportion of severe erosion decreased from 22.54% to 14.14%, indicating that the soil erosion situation had been greatly improved.

### 3.4. Changes in Soil Erosion before and after Vegetation Restoration

**Figure 4:**
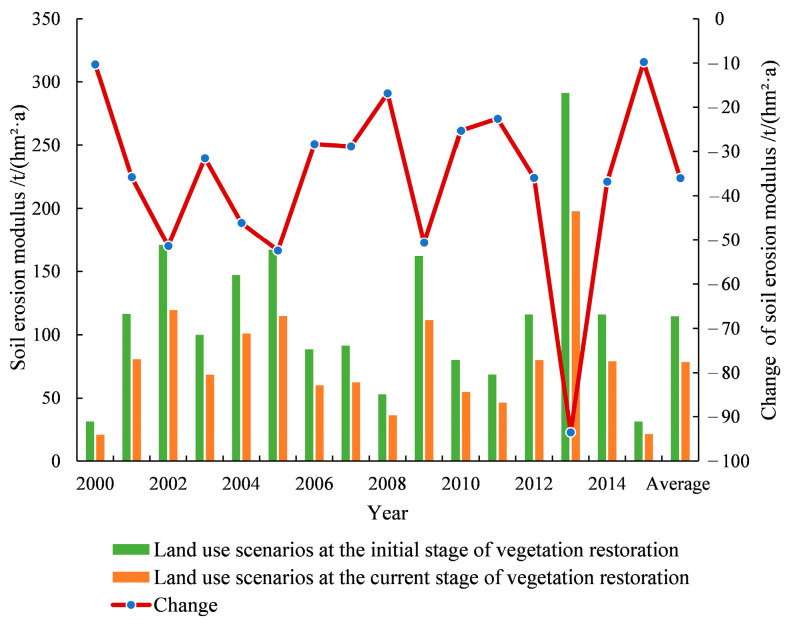
Effects of vegetation restoration on soil erosion from 2000 to 2015.

The average soil erosion modulus from 2000 to 2015 under the initial and the current scenarios of vegetation restoration was 114.44 t/(hm^2^·a) and 78.42 t/(hm^2^·a), respectively, with an average annual reduction of 4.81 × 10^6^ t of soil erosion amount. The soil erosion condition was improved by vegetation restoration, and the soil erosion modulus decreased annually by 10.30, 35.78, 51.37, 31.51, 46.14, 52.40, 28.39, 28.87, 16.87, 50.62, 25.31, 22.61, 35.96, 93.51, 36.83, and 9.75 t/hm^2^ from 2000 to 2015, respectively (Figure 4). In addition, the effects of vegetation restoration on soil erosion were different in different years, mainly because of the large differences of rainfall in each year. The water and soil conservation benefits of vegetation restoration was more obvious in the years with heavy rainfall such as 2002, 2005, 2009, and 2013.

**Figure 5:**
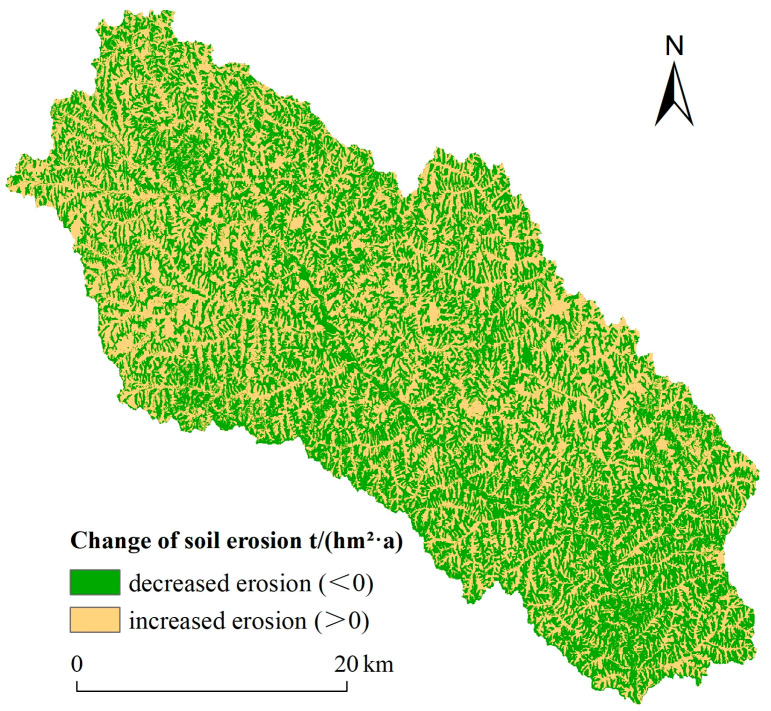
Spatial differentiation of the effect of vegetation restoration on soil erosion in 2000–2015.

The average soil erosion modulus changes from 2000 to 2015 were divided into two categories, increased erosion (>0) and decreased erosion (<0), based on the reclassification function of ArcGIS 10.6 (Figure 5). During the study period, the areas with increased erosion and decreased erosion were alternately distributed in the Ansai Watershed. The south and southeast of the Ansai Watershed had obvious improvement effects on soil erosion and were the main areas with decreased soil erosion, while the northwest of the study area was the main region experiencing increased soil erosion. Although the areas with increased and decreased soil erosion distributed alternatively, the former was lower than the latter (Table 6). The area of decreased and increased soil erosion from 2000 to 2015 was 696.92 km^2^ and 637.12 km^2^, respectively, accounting for 52% and 48% of the total land area of the Ansai Watershed.

## 4. Discussion

### 4.1. Effects of Vegetation Restoration on Soil Erosion

Land use types not only affect the properties of the underlying soil surface, but also influence the redistribution of rainfall and the transport of runoff and sediment [46]. According to previous research in Yanan city, the soil conservation modulus varied with the land use types; furthermore, forest land and grassland had the best soil conservation effects [47]. Since the implementation of the “GGP” in 1999, the land use structure in the Ansai Watershed has undergone significant changes, mainly characterized by the conversion of sloping farmland to grassland, arboreal land, and shrub land. The effective implementation of this project significantly improved the soil erosion environment in the study area, in accordance with previous research results [40,48,49]. Wang et al. (2016) [49] found that compared with the sloping farmland, the conversion of sloping farmland to grassland or woodland can reduce gully erosion by more than 90%. Results of this study indicated that the average annual soil erosion modulus dropped from 114.56 t/(hm^2^·a) to 78.42 t/(hm^2^·a), and the dominant soil erosion intensity changed from severe erosion and light erosion to moderate erosion and light erosion in the Ansai Watershed during 2000–2015. In addition, according to the data released by the China National Forestry and Grassland Administration (http://www.forestry.gov.cn/ accessed on 25 April 2021), the average annual soil erosion modulus in the Ansai County dropped from 140.00 t/(hm^2^·a) in 1998 to 54.00 t/(hm^2^·a) in 2018 since the implementation of the “GGP”, which further confirmed the accuracy and credibility of our research results. By the end of 2018, a total of 94,920 hm^2^ forest land was increased, of which 56,520 hm^2^ was transferred from sloping farmland, and 36,470 hm^2^ was transferred from desert land and grassland in Ansai County [50]. Thanks to massive vegetation restoration, with increasing vegetation coverage and biomass, the dense vegetation canopy reduced the effective precipitation in forest land, prolonged the precipitation and runoff duration, and cut off the kinetic energy of raindrops; surface mulch dispersed the kinetic energy of runoff, and the complex vegetation root system increased the resistance of the soil runoff erosion, effectively strengthened the regional soil and water conservation benefits, and improved soil erosion conditions [51]. Furthermore, previous studies showed that the soil profile structure destroyed by erosion became more and more complete, and soil properties were restored in loess hilly and gully regions after the implementation of “GGP” [16]. For example, soil bulk density and PH value decreased, while soil organic matter content, C, and N content increased. The conversion of sloping farmland to forest land with relatively little human interference was conducive to the accumulation of soil nutrients and the maintenance of porosity, and effectively enhanced the water and fertilizer retention performance of soil.

The spatial differentiation of soil erosion changes also further indicated the positive effect of vegetation restoration on soil erosion. Vegetation restoration was actively carried out in the southeast and south of the Ansai Watershed, where the land use change was relatively drastic, and mainly included transformations from farmland to grassland, shrub land and arboreal land, and from grassland to shrub land and arboreal land. The massive vegetation restoration in this area effectively strengthened the water and soil conservation benefits, and greatly improved the soil erosion condition. However, in the northwest of the Ansai Watershed, the implementation effect of vegetation restoration was poor, which led to more serious soil erosion in local areas.

### 4.2. Policy Implications

The main reason for the poor soil erosion control effect in the northwest of the Ansai Watershed was the ineffective implementation of the “GGP” and the unreasonable land use changes, such as the conversion of farmland and grassland into construction land. In view of this, local governments should actively carry out this project, strictly implement land use planning, control the occupation of farmland for non-agricultural construction, and prevent unreasonable land use changes.

The field survey in the Ansai Watershed found that although the “GGP” was also carried out in the northwest of the watershed and a certain number of sea-buckthorn and Caragana korshinskii plants were planted in this region where soil erosion was increasingly serious, the majority of these trees did not survive due to lack of supervision and management. Previous studies showed that the phenomenon of “seeing only the saplings but not the forest” was common in the process of returning farmland to forest in Ansai County [48]. The main reasons include the following two aspects: first, farmers lacked the initiative in the management of forest seedlings, and only focused on the subsidies for returning farmland to forest, but ignored the follow-up management of forest land; second, the lack of support for pest control and forest fire prevention directly affected the quality and subsequent benefits of this project. The implementation of the “GGP” is a long-term process, and the local government should strengthen the supervision and management of ecological restoration, follow the principle of “whoever builds, manages and benefits”, strengthen the management and protection of vegetation seedlings, and ensure the normal growth of young forests. Considering the fragile ecological environment in the Loess Plateau region, excellent tree and grass species with strong adaptability and good quality should be selected to improve the survival rate of seedlings. Local governments should carry out inspections of vegetation restoration occasionally. To ensure the effects of vegetation restoration, measures such as supplementary planting, tending, pruning, watering, weeding, and pest control should be taken for forest land converted from farmland with substandard numbers of living plants and low survival rates.

### 4.3. Research Limitation and Future Research

The CSLE model proposed by the Chinese scholar is widely used to calculate the soil erosion amount in China. The applicability of this model in Ansai county, the loess hilly and gully regions, and even in China has been verified by previous studies [32,33,52,53,54,55,56], and can well reflect the soil erosion situation in China. Among them, by comparing the simulation results of the CSLE model with the soil erosion data measured by the Ministry of Water Resources of the People’s Republic of China, the predecessors proved that the model has good applicability and credibility in the soil erosion evaluation in Shaanxi Province [52]. In view of this, this study assessed the soil erosion amount using the CSLE model in the Ansai Watershed belonging to Shaanxi Province. Furthermore, all parameters of the CSLE model in this study were calibrated based on previous research. Although model uncertainties are unavoidable, the calculation results of the soil erosion modulus in the Ansai watershed using the CSLE model can reflect the actual situation.

Unlike the soil erosion amount which can be measured on the field spot, the effect of vegetation restoration on soil erosion cannot be directly measured through field experiments. Through field control experiments, comparing the changes of soil erosion amount in the two watersheds with the same conditions only, except for implementing or not implementing the “GGP”, can verify our research results to a certain extent. However, considering the effect of vegetation restoration on soil erosion is correlated with the length of time for reforestation [20], short-term field monitoring results through field control experiments cannot verify the model simulation results from 2000 to 2015. Furthermore, it takes a lot of manpower and time to conduct continuous monitoring of soil erosion for more than 10 years at the watershed scale, and the gap in existing data sources also makes it difficult to currently verify our research results on the field spot. Under the above constraints, we validated our research results based on the official data on the effect of vegetation restoration on soil erosion in Ansai County released by the National Forestry and Grass Administration of the People’s Republic of China, and the previous research results in loess hilly and gully regions. It is worth mentioning that the data released by the National Forestry and Grass Administration was obtained through on-site monitoring of the Ansai Hydrological Station, which is not the model simulation result, and its consistency with our research results provided a good proof of the credibility of our research. In follow-up studies, we will increase long-term field monitoring experiments to more accurately assess the effect of vegetation restoration on soil erosion.

## 5. Conclusions

Large-scale vegetation restoration triggered by the “Grain for Green Project” (GGP) since 1999 led to significant land use changes in the Loess Plateau. Using the CSLE model, this study calculated and compared the differences between the soil erosion modulus from 2000 to 2015 under two land use scenarios (the initial and current scenarios of vegetation restoration), and identified the effect of vegetation restoration on soil erosion in the Ansai Watershed. The results showed that the soil erosion conditions have greatly improved in the Ansai Watershed since vegetation restoration. The average soil erosion modulus under the initial scenario of vegetation restoration among the 16 years was 114.56 t/(hm^2^·a), dominated by severe erosion and light erosion; while the average soil erosion modulus under the current scenario of vegetation restoration among the 16 years was 78.42 t/(hm^2^·a), with light and moderate erosion as the dominant soil erosion intensity. However, due to the unreasonable land use changes (farmland, grassland was converted into construction land, etc.) and the ineffective implementation of vegetation restoration, soil erosion became more serious in some areas. Therefore, it is necessary to strengthen the supervision and management of the “GGP”, control unreasonable land use changes by land use planning, and prevent decreases in vegetation coverage to control soil erosion in the Ansai Watershed.

## Figures and Tables

**Table 1:** *B* factor under different land use types and different vegetation coverage.

Land Use Type	Vegetation Coverage (%)	*B* Factor	Land Use Type	Vegetation Coverage (%)	*B* Factor
Arboreal and shrub land	0~20	0.100	Grassland	0~20	0.450
	20~40	0.080		20~40	0.240
	40~60	0.060		40~60	0.150
	60~80	0.020		60~80	0.090
	80~100	0.004		80~100	0.043
Water	–	0.000	Farmland	–	0.476
Construction land	–	0.353	Desert land	–	1.000

**Table 2:** *T* factor under different slope gradient.

Slope Gradient	≤5°	5–10°	10–15°	15–20°	20–25°	>25°
*T* factor	0.100	0.221	0.305	0.575	0.735	0.800

**Table 3:** Transfer matrix of land use changes in the Ansai Watershed from 2000 to 2015 (km^2^).

Land Use Type		2015							Total
		Arboreal Land	Shrub Land	Grassland	Farmland	Construction Land	Water	Desert Land	
2000	Arboreal land	2.41	1.20	16.01	0.04	0.14	0.16	0.01	19.96
	Shrub land	1.22	16.07	2.00	0.07	0.02	0.04	0.01	19.43
	Grassland	44.83	23.43	643.08	1.66	2.13	3.13	1.24	719.49
	Farmland	50.72	35.25	422.78	32.57	3.84	1.86	0.89	547.90
	Construction land	0.03	0.02	0.18	0.02	1.89	0.01	0.00	2.14
	Water	0.04	0.01	0.20	0.00	0.00	0.00	0.00	0.25
	Desert land	0.00	0.00	0.00	0.00	0.00	0.00	0.00	0.00
Total		99.26	75.97	1084.24	34.35	8.02	5.20	2.14	–
Change from 2000 to 2015		79.30	56.54	364.75	−513.55	5.88	4.95	2.14	–

**Table 4:** Soil erosion in the Ansai Watershed from 2000 to 2015 under the initial scenario of vegetation restoration.

Year	Soil Erosion Modulus (t/(hm^2^·a))	Area of Different Soil Erosion Intensity (%)					
		Slight	Light	Moderate	Serious	Extreme	Severe
2000	31.18	22.88	41.67	15.47	8.51	8.86	2.60
2001	116.45	8.18	18.44	18.64	13.75	16.19	24.80
2002	170.88	6.12	13.65	14.53	13.15	18.39	34.16
2003	99.92	9.55	21.30	19.14	13.77	14.60	21.63
2004	147.21	7.21	15.29	16.66	13.20	17.45	30.19
2005	167.17	6.59	13.96	15.02	13.09	17.97	33.36
2006	88.56	10.69	23.77	19.44	13.11	13.90	19.09
2007	91.38	10.25	22.74	19.37	13.52	14.23	19.88
2008	55.03	14.68	33.43	19.72	10.53	11.42	10.23
2009	162.21	6.74	14.46	15.26	13.31	17.67	32.55
2010	80.11	11.15	25.45	19.80	12.87	13.49	17.24
2011	68.66	15.07	29.23	18.47	11.36	12.02	13.85
2012	115.97	8.52	18.78	18.40	13.71	15.92	24.67
2013	291.11	4.45	9.45	8.81	10.59	17.53	49.16
2014	115.96	8.61	18.41	18.64	13.74	15.75	24.85
2015	31.19	22.34	41.79	15.98	8.41	9.10	2.38
Average	114.56	10.81	22.61	17.09	12.29	14.66	22.54

Note: Slight erosion (≤5 t/(hm^2^·a)), light erosion (5–25 t/(hm^2^·a)), moderate erosion (25–50 t/(hm^2^·a)), serious erosion (50–80 t/(hm^2^·a)), extreme erosion (80–150 t/(hm^2^·a)), and severe erosion (>150 t/(hm^2^·a)).

**Table 5:** Soil erosion in the Ansai Watershed from 2000 to 2015 under the current scenario of vegetation restoration.

Year	Soil Erosion Modulus (t/(hm^2^·a))	Area of Different Soil Erosion Intensity (%)					
		Slight	Light	Moderate	Serious	Extreme	Severe
2000	20.88	22.16	49.11	20.38	5.63	2.28	0.44
2001	80.67	7.82	18.43	19.00	18.51	22.07	14.16
2002	119.51	5.85	13.72	13.52	15.16	24.09	27.67
2003	68.41	8.93	20.98	21.86	18.03	20.51	9.69
2004	101.07	6.86	15.22	15.58	17.19	23.53	21.62
2005	114.77	6.19	13.98	13.84	15.71	24.07	26.22
2006	60.17	9.91	23.61	23.74	17.38	18.12	7.23
2007	62.51	9.42	22.58	23.50	17.54	19.26	7.70
2008	36.16	14.47	34.98	26.34	14.77	7.32	2.12
2009	111.59	6.16	14.84	14.87	15.96	23.49	24.67
2010	54.80	10.38	25.14	25.15	17.16	16.68	5.50
2011	46.05	14.21	31.27	23.00	14.28	12.81	4.44
2012	80.01	7.87	19.09	19.71	17.85	21.54	13.93
2013	197.60	4.22	9.32	8.86	9.85	21.00	46.75
2014	79.13	7.94	18.72	19.33	18.50	21.91	13.60
2015	21.44	22.05	48.37	20.87	5.78	2.37	0.56
Average	78.42	10.28	23.71	19.35	14.96	17.57	14.14

Note: Slight erosion (≤5 t/(hm^2^·a)), light erosion (5–25 t/(hm^2^·a)), moderate erosion (25–50 t/(hm^2^·a)), serious erosion (50–80 t/(hm^2^·a)), extreme erosion (80–150 t/(hm^2^·a)), and severe erosion (>150 t/(hm^2^·a)).

**Table 6:** Changes in soil erosion areas and proportions from 2000 to 2015.

Year	Increased Erosion Area (km^2^)	Proportion (%)	Decreased Erosion Area (km^2^)	Proportion (%)
2000	630.34	47.25	703.66	52.75
2001	673.74	50.51	661.73	49.60
2002	625.30	46.87	707.83	53.06
2003	634.18	47.54	699.67	52.45
2004	614.26	46.05	719.81	53.96
2005	614.26	46.05	719.81	53.96
2006	640.90	48.04	693.10	51.96
2007	642.82	48.19	691.18	51.81
2008	640.46	48.01	693.54	51.99
2009	640.81	48.04	693.19	51.96
2010	638.50	47.86	695.50	52.14
2011	640.93	48.05	693.06	51.95
2012	638.06	47.83	695.94	52.17
2013	653.53	48.99	680.47	51.01
2014	637.94	47.82	696.06	52.18
2015	627.85	47.07	706.14	52.93
Average	637.12	47.76	696.92	52.24

## Data Availability

All relevant data sets in this study are described in the manuscript.
